# IL-7 Promotes the Expansion of Circulating CD28- Cytotoxic T Lymphocytes in Patients With IgG4-Related Disease *via* the JAK Signaling

**DOI:** 10.3389/fimmu.2022.922307

**Published:** 2022-07-07

**Authors:** Chang-sheng Xia, Yan Long, Yanying Liu, Aikede Alifu, Xingyue Zeng, Chen Liu

**Affiliations:** ^1^ Department of Clinical Laboratory, Peking University People’s Hospital, Beijing, China; ^2^ Department of Rheumatology and Immunology, Beijing Friendship Hospital, Capital Medical University, Beijing, China

**Keywords:** IgG4-related disease, cytotoxic T lymphocytes, interleukin-7, CD28, tofacitinib

## Abstract

**Objectives:**

This study aimed to elucidate the changes and associated mechanisms of circulating CD28- cytotoxic T lymphocytes (CTLs) in patients with IgG4-related disease (IgG4-RD).

**Methods:**

Fifty IgG4-RD patients and 15 healthy controls (HCs) were recruited. Peripheral blood mononuclear cells (PBMCs) were isolated, the levels of circulating CD28- CTLs were detected by flow cytometry, and the proportions of CD127lo or GZMB+CD28- CTL subsets were analyzed in the meantime. Mechanistically, PBMCs isolated from IgG4-RD patients were stimulated with IL-7 in the presence or absence of the JAK inhibitor tofacitinib. Flow cytometry was used to analyze the proliferation of CD28- CTLs and the changes in related subpopulations.

**Results:**

Circulating CD4+CD28- CTLs and CD8+CD28- CTLs were significantly increased in IgG4-RD patients compared with HCs, accompanied by an elevation of CD127lo or GZMB+ CTL subsets. The *ex vivo* culture of PBMCs showed that IL-7 could induce the amplification of CD4+CD28- CTLs and CD8+CD28- CTLs in IgG4-RD. Furthermore, IL-7 promotes the proliferation and functional subset changes of these CD28- CTLs in this disease. The selective JAK inhibitor tofacitinib significantly inhibited the effects of IL-7 on CD4+CD28- CTLs and CD8+CD28- CTLs.

**Conclusion:**

IL-7 can affect the immune balance of IgG4-RD patients by promoting the expansion and function of CD4+CD28- and CD8+CD28- CTLs in IgG4-RD through the JAK pathway. Blockade of the IL-7 signaling pathway may be a new therapeutic strategy for IgG4-RD.

## Highlights

-Circulating CD28- CTLs were significantly elevated in patients with IgG4-RD.
-IL-7 promotes the expansion and function of CD28- CTLs in IgG4-RD patients.
-The effects of IL-7 are significantly inhibited by the JAK inhibitor tofacitinib.

## Introduction

As one newly discovered chronic fibroinflammatory disease, IgG4-related disease (IgG4-RD) is characterized by frequently elevated serum IgG4 concentrations, infiltration of dense lymphoplasmacytic cells in the lesions, and favorable response to glucocorticoid therapy (1–3). Most infiltrating lymphocytes are CD4 T cells and CD8 T cells (4, 5). These findings strongly suggest that T lymphocytes may play a crucial role in the development of this disease.

Many studies have focused on T cell subsets to identify the immune mechanisms of this disease, of which cytotoxic T lymphocytes (CTLs) have attracted increasing attention (6, 7). CD28 negative T lymphocytes, including CD4+CD28- T cells and CD8+CD28- T cells, have been reported to produce inflammatory cytokines and cytotoxic molecules (6, 7). It had shown that such CTLs are clonally expanded in the lesions and blood of IgG4-RD patients and are involved in the pathogenesis of the disease (5). However, the exact mechanisms driving CD28- CTL expansion in IgG4-RD patients remains undefined, and the changes in levels of CD28- CTL-associated subsets have not been fully elucidated.

Interleukin-7 (IL-7), a 25 kDa secreted soluble globular protein, plays an irreplaceable role in T cell development and homeostasis (8–11). Its receptor (IL-7R) is a heterodimer comprised of IL-7Rα (CD127) and the common cytokine receptor γ-chain (CD132), shared with the receptors for IL-2, IL-4, IL-9, IL-15, and IL-21. In all T cells, IL-7/IL-7R initiates downstream signaling pathways mainly through the activation of the Janus kinase–signal transducer and activator of transcription pathway (JAK-STAT pathway) to increase T cell survival and proliferation (12). Because the expression of the CD132 is ubiquitous on T cells, the effects of IL-7 are regulated by the expression of the CD127, likewise by the availability of the cytokine IL-7 itself (13). A previous study showed that CD8+CD28- CTLs were selectively expanded when activated CD8+CD28+ T cells were cultured with IL-7 in healthy individuals (14). Recent research reported that IL-7 could enhance the expansion and function of CD4+CD28- CTLs in patients with acute coronary syndrome (15). In addition, the researchers demonstrated that IL-7 was up-regulated in lesion tissues from patients with IgG4-RD (16, 17). But whether IL-7 is involved in the expansion and function of CD28- CTLs in IgG4-RD remains unknown.

In this study, we analyzed the changes of CD4+CD28- CTLs and CD8+CD28- CTLs and their related subsets in the circulation of IgG4-RD patients by flow cytometry. We further investigated the effects of IL-7 on CD28- CTLs in IgG4-RD patients and inquired into the molecular mechanisms.

## Materials and Methods

### Subjects

A total of 50 patients with IgG4-RD and 15 healthy controls (HCs) were recruited from outpatient and inpatient sections of Peking University People’s Hospital between March 2021 and Jan 2022. The diagnosis of IgG4-RD was performed according to the 2011 comprehensive diagnostic criteria (18). Forty-seven IgG4-RD patients received therapy with glucocorticoids alone or in combination with immunosuppressive agents. The mean number of involved organs was 3.6 in IgG4-RD patients. The involvement organs included the submandibular glands (38 cases), lacrimal glands (33 cases), lymph nodes (24 cases), pancreas (18 cases), parotid gland (13 cases), bile ducts (13 cases), kidney (10 cases), skin (8 cases), retroperitoneum (6 cases), lung (6 cases), sinuses (3 cases), gallbladder (2 cases), sublingual gland (1 case), prostate (1 case), adrenal gland (1 case), hypophysis (1 case), and aorta (1 case). The IgG4-RD responder index (RI) score was calculated and used for the assessment of disease activity (19). Briefly, all individual organ scores and serum IgG4 scores were summed to calculate the total activity score. This research was approved by the Ethics Committee of Peking University People’s Hospital and was performed following the ethical standards of the Declaration of Helsinki.

### Clinical Indicator Measurement

Serum levels of IgG4 were measured by nephelometry using a Siemens BN II Nephelometer (Siemens Healthcare Diagnostics; Malburg, Germany) and Siemens reagents. Serum IgE levels were tested by a Cobas e601 Electrochemiluminescence Immunoassay Analyzer (Roche; Mannheim, Germany).

### PBMCs Isolation

Peripheral blood mononuclear cells (PBMCs) were separated from fresh blood samples by gradient centrifugation with a human lymphocyte separation medium (Hao Yang; Tianjin, China) and then washed twice with PBS.

### Cell Culture

Enriched PBMCs were resuspended in RPMI 1640 (Solarbio; Beijing, China), supplemented with 10% FBS. Cells were stimulated with recombinant human IL-7 (50 ng/mL) (Beyotime; Shanghai, China) in the presence or absence of 150 nM tofacitinib (Meilun Biotechnology; Dalian, China) and cultured for up to 4 days in 96-well plates.

### Flow Cytometry

Fresh or cultured PBMCs were stained with antibodies against surface markers, including CD3-APC, CD4-PE/Cy7, CD8-APC/Cy7, CD28-PE, CD127-PerCP/Cy5.5. Then intracellular staining was performed using a transcription factor staining buffer kit (Thermo Fisher Scientific-eBioscience; San Diego, CA, USA), according to the manufacturer’s instructions. After fixation and permeabilization, cells were incubated with antibodies against intracellular markers including GZMB (granzyme B)-FITC and Ki67-PerCP/Cy5.5 for 30 minutes. The mean fluorescence intensity (MFI) of GZMB was calculated. All fluorescent antibodies were purchased from BioLegend (San Diego, CA, USA). Samples were analyzed on FACSCanto using Diva software (BD Biosciences; San Jose, CA, USA).

### Statistics

Categorical variables were analyzed with a χ^2^ test and were shown as percentages. Continuous variables were presented as mean with SD or median with an interquartile range. The unpaired two-tailed Student’s t-test or two-tailed Mann–Whitney test was used for the comparison of two groups as indicated. Cytokine-treated and untreated samples were compared using paired two-tailed Student’s t-test. Statistical significance was determined using GraphPad Prism software V.7.0 (GraphPad Software; San Diego, CA, USA). Statistics with *P* values less than 0.05 were considered to be significant.

## Results

### The Levels of Circulating CD4+CD28- CTLs and CD8+CD28- CTLs Were Significantly Elevated in IgG4-RD Patients

Totally 50 patients and 15 healthy controls were enrolled in this study. The demographics and the levels of serum IgG4 and IgE were listed in **Table 1**. The concentrations of serum IgG4 were significantly higher in IgG4-RD patients than those in HCs (3.51 g/L versus 0.38 g/L; *P* < 0.0001). Similarly, IgE levels were significantly elevated in IgG4-RD patients compared to HCs (176.0 IU/mL versus 26.7 IU/mL; *P* < 0.0001).

**Table 1:** The demographics and the levles of serum IgG4 and IgE in IgG4-RD patients and healthy controls.

Variable	IgG4-RD	HC	*P* values
**No.**	50	15	
**Male, % (n)**	64 (32)	60 (9)	0.778^*^
**Age, years, mean (SD)**	60 (10)	57 (8)	0.361^†^
**IgG4, g/L, median (IQR)**	3.51 (4.87)	0.38 (0.60)	< 0.0001^‡^
**IgE, IU/mL, median (IQR)**	176.0 (275.2)	26.7 (45.2)	< 0.0001^‡^

*Categorical variables were analyzed with a χ^2^ test. ^†^the unpaired Student’s t-test was performed to calculate two-sided P values. ^‡^the Mann-Whitney test was used to calculate two-sided P values.

**Figure 1:**
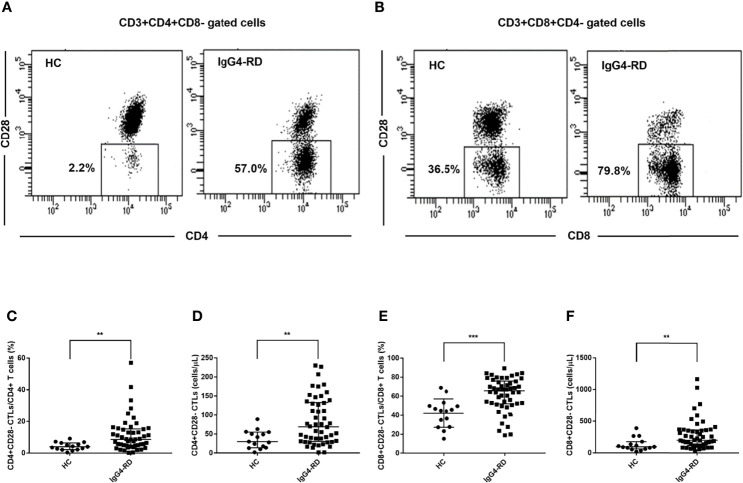
Analysis of the levels of CD28- CTLs in peripheral blood from IgG4-RD patients. Peripheral blood mononuclear cells from IgG4-RD patients (*n* = 50) and healthy controls (HCs, *n* = 15) were extracted and analyzed by flow cytometry for the levels of CD4+CD28- and CD8+CD28- CTLs in CD4 and CD8 T cells. **(A, B)** Representative dot plots of flow cytometry analysis. CD4+CD28- CTLs and CD8+CD28- CTLs were gated. **(C–F)** The levels of circulating CD4+CD28- CTLs and CD8+CD28- CTLs were compared between IgG4-RD patients and HCs. The error bars represented the median with an interquartile range. The two-tailed Mann-Whitney test was used to compare two groups. ^**^ *P* < 0.01;^∗∗∗^ *P* < 0.001.

We analyzed CD4+CD28- CTLs and CD8+CD28- CTLs in the blood of patients with IgG4-RD and age-matched HCs by flow cytometry. We observed a significant increase in the levels of CD4+CD28- CTLs and CD8+CD28- CTLs in IgG4-RD patients (**Figure 1**).

We further analyzed the correlations between the levels CD4+CD28- CTLs or CD8+CD28- CTLs and IgG4-RD RI or the concentrations of serum IgG4 and IgE. No significant correlation was found between them (**Supplementary Figures 1**, **2**).

### Circulating CD4+CD28-CD127LO CTLs and CD8+CD28-CD127LO CTLs Were Increased in IgG4-RD Patients

**Figure 2:**
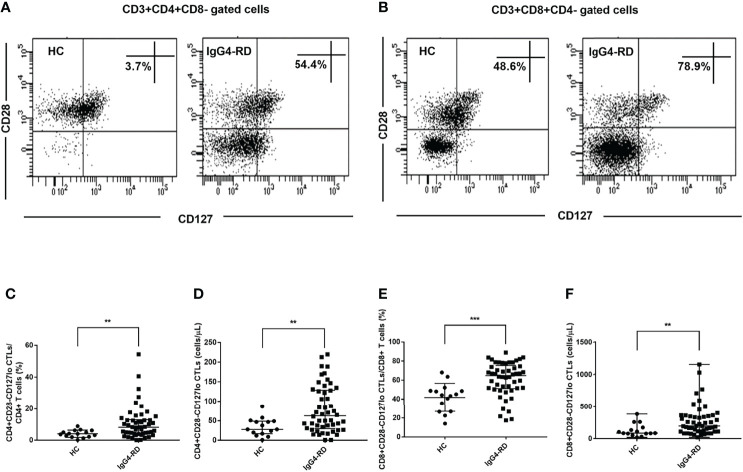
Analysis of the levels of CD28-CD127lo CTLs in CD4 and CD8 T cells. CD4+CD28-CD127lo and CD8+CD28-CD127lo CTLs in the circulation of IgG4-RD patients (n = 50) and healthy controls (HCs, n = 15) were analyzed using flow cytometry. **(A, B)** Representative dot plots of flow cytometry analysis. CD4+CD28-CD127lo and CD8+CD28-CD127lo CTL percentages were indicated. CD127 was gated according to fluorescence minus one (FMO) control of CD127. **(C–F)** The levels of circulating CD4+CD28-CD127lo CTLs and CD8+CD28-CD127lo CTLs were compared between IgG4-RD patients and HCs. The error bars represented the median with an interquartile range. The two-tailed Mann-Whitney test was used to compare two groups. ^∗∗^ *P* < 0.01;^∗∗∗^ *P* < 0.001.

We further analyzed the changes of CD28-CD127lo CTLs in peripheral blood of IgG4-RD patients and HCs. Similarly, the levels of CD4+CD28-CD127lo CTLs and CD8+CD28-CD127lo CTLs were significantly higher in patients with IgG4-RD compared with HCs (**Figure 2**). This indicated that many of the increased CD4+CD28-CTLs and CD8+CD28- CTLs in IgG4-RD patients were CD127lo cells.

### CD4+CD28-GZMB+ CTLs and CD8+CD28-GZMB+ CTLs Were Increased in the Circulation of IgG4-RD Patients

**Figure 3:**
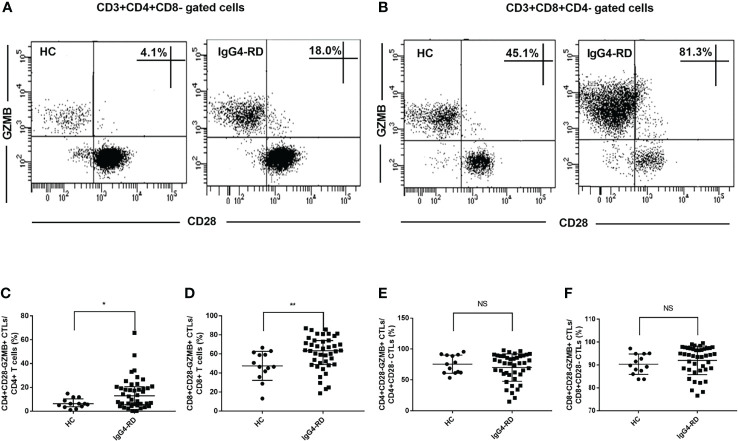
Analysis of the levels of CD28-GZMB+ CTLs in peripheral blood from IgG4-RD patients and healthy controls. Peripheral blood mononuclear cells of IgG4-RD patients (*n* = 42) and healthy controls (HCs, n = 13) were obtained, CD3, CD4, CD8, CD28 were detected by flow cytometry and GZMB was detected by intracellular staining. **(A, B)** Representative dot plots of flow cytometry analysis. CD4+CD28-GZMB+ CTLs and CD8+CD28-GZMB+ CTLs were shown. **(C, D)** The percentages of circulating CD4+CD28-GZMB+ CTLs and CD8+CD28-GZMB+ CTLs in CD4 and CD8 T cells were compared between IgG4-RD patients and HCs. **(E, F)** The proportions of GZMB+ cells in CD4+CD28- CTLs and CD8+CD28- CTLs were compared. The error bars represented the median with an interquartile range. The two-tailed Mann–Whitney test was used to compare two groups. ^∗^ *P* < 0.05; ^∗∗^ *P* < 0.01; NS, not significant.

GZMB is one of the most important effector molecules of cytotoxic T cells. We used CD28 surface molecules to characterize CTLs, and we need to clarify the expression of GZMB inside them. As shown in **Figures 3A, B**, the majority of CD28- CTLs expressed the cytotoxic marker GZMB. We analyzed the levels of CD4+CD28-GZMB+ CTLs and CD8+CD28-GZMB+ CTLs and found that the percentages of CD4+CD28-GZMB+ CTLs and CD8+CD28-GZMB+ CTLs were higher in IgG4-RD patients than in HCs (**Figures 3C, D**). We also analyzed GZMB+ cell percentages in CD4+CD28- CTLs and CD8+CD28- CTLs and found that the mean percentage reached over 70% in CD4+CD28- CTLs and over 90% in CD8+CD28- CTLs, but did not reach a significant difference level between IgG4-RD patients and HCs (**Figures 3E, F**).

### CD4+CD28- CTLs and CD8+CD28- CTLs of IgG4-RD Patients Expanded After Co-culture With IL-7 Could be Inhibited by Adding Tofacitinib

**Figure 4:**
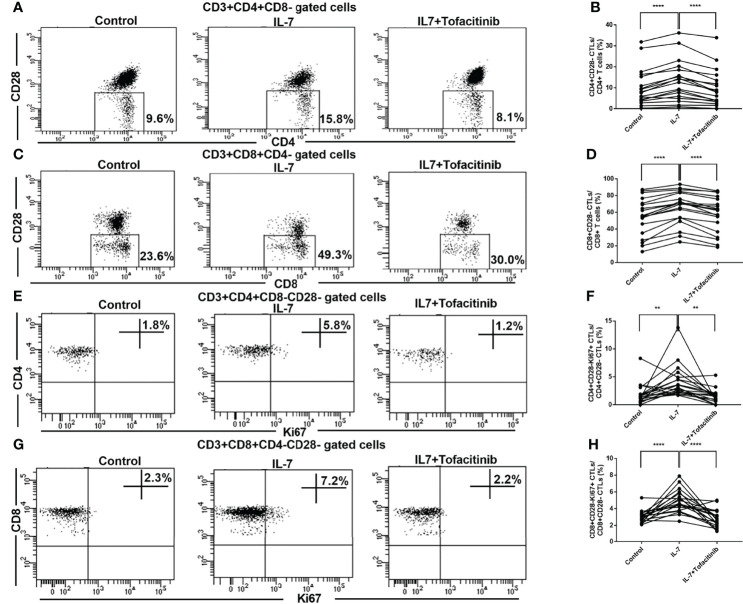
Expression and activation analysis of CD28- CTLs in peripheral blood from IgG4-RD patients with the addition of IL-7 and JAK inhibitor. PBMCs extracted from IgG4-RD patients were cultured with 50 ng/mL IL-7 in the presence or absence of 150 nM JAK inhibitor tofacitinib for 4 days. **(A, C)** Representative dot plots of flow cytometry analysis. CD4+CD28- and CD8+CD28- CTL percentages in CD4 and CD8 T cells were indicated. **(B, D)** The percentages of CD4+CD28- CTLs and CD8+CD28- CTLs in IgG4-RD patients (n = 19) were compared between IL-7-treated and untreated samples, and between IL-7-treated and IL-7 plus tofacitinib samples as well. The paired two-tailed Student’s t-test was used to compare two groups. ****P< 0.0001. **(E, G)** Representative dot plots of flow cytometry analysis. Ki67+ cell percentages in CD4+CD28- and CD8+CD28- CTLs were shown. Ki67 was gated according to fluorescence minus one (FMO) control of Ki67, and Ki67 staining after PBMC stimulation with PMA for 6 hours was used as a positive control for gating. **(F, H)** Ki67+ cell percentages in CD4+CD28- CTLs and CD8+CD28- CTLs in IgG4-RD patients (n = 19) were compared between IL-7-treated and untreated samples, and between IL-7-treated and IL-7 plus tofacitinib samples as well. The paired two-tailed Student’s t-test was used to compare two groups. **P< 0.01; ****P< 0.0001.

We next investigated the effects of IL-7 on CD28- CTLs from IgG4-RD patients and analyzed the role of JAK signaling downstream of IL-7. PBMCs from IgG4-RD patients were treated with IL-7 in the presence or absence of tofacitinib. The percentages of CD4+CD28- CTLs and CD8+CD28- CTLs were significantly increased by the stimulation of IL-7 (**Figures 4A–D**). Meanwhile, the increase could be significantly inhibited by tofacitinib (**Figures 4A–D**). We investigated the proliferation of CTLs by intracellular staining of Ki-67. The percentages of CD4+CD28-Ki-67+ CTLs and CD8+CD28-Ki-67+ CTLs were increased significantly by co-culture with IL-7 (**Figures 4E–H**). And these effects could also be significantly inhibited by adding tofacitinib (**Figures 4E–H**).

### CD8+CD28-CD127lo CTLs of IgG4-RD Patients Were Expanded Upon Addition of IL-7 and Recovered Upon Addition of JAK Inhibitor

**Figure 5:**
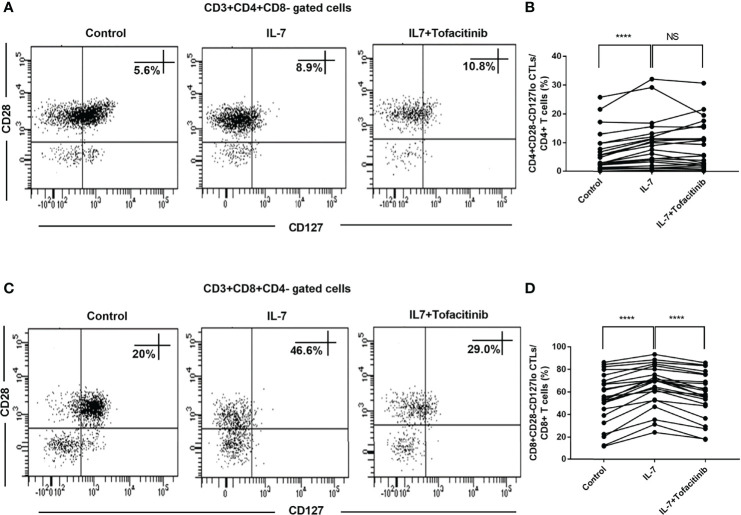
IL-7 induced elevated levels of CD8+CD28-CD127lo CTLs inhibited by JAK inhibitor in IgG4-RD patients. PBMCs from IgG4-RD patients (n = 23) were cultured with 50 ng/mL IL-7 in the presence or absence of 150 nM tofacitinib for 4 days. **(A, C)** Representative dot plots of flow cytometry analysis. CD4+CD28-CD127lo and CD8+CD28-CD127lo CTL percentages in CD4 and CD8 T cells were shown. CD127 was gated according to fluorescence minus one (FMO) control of CD127. **(B, D)** The percentages of CD4+CD28-CD127lo CTLs and CD8+CD28-CD127lo CTLs in IgG4-RD patients were compared between IL-7-treated and untreated samples, and between IL-7-treated and IL-7 plus tofacitinib samples as well. The paired two-tailed Student’s t-test was used to compare two groups. ****P< 0.0001; NS, not significant.

We further analyzed the changes of CD28-CD127lo CTLs after adding IL-7. We found that both CD4+CD28-CD127lo CTLs and CD8+CD28-CD127lo CTLs were expanded after co-culture with IL-7 (**Figures 5A–D**). The role of the JAK pathway was verified by adding tofacitinib for CD8+CD28-CD127lo CTLs, and the results showed that the addition of tofacitinib could reduce the expansion of CD8+CD28-CD127lo CTLs rather than CD4+CD28-CD127lo CTLs caused by the addition of IL-7 (**Figures 5A–D**).

### IL-7 Could Increase CD28-GZMB+ CTL Proportions in IgG4-RD Patients and Up-Regulate GZMB Expression Through the JAK Pathway

**Figure 6:**
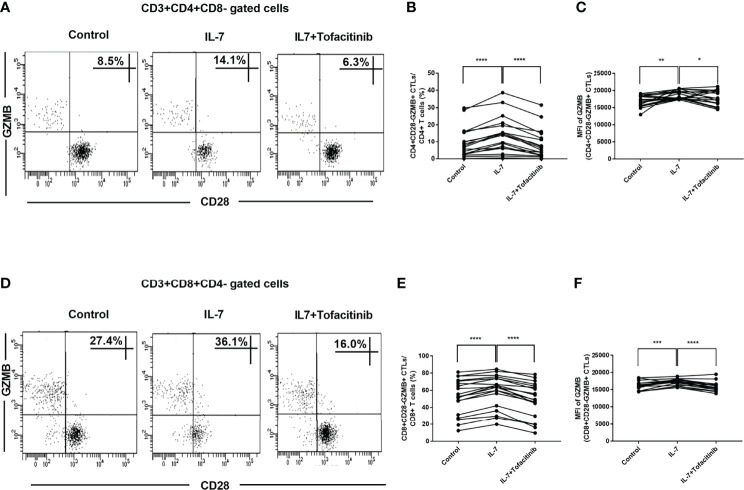
The effects of IL-7 on the proportions of CD28-GZMB+ CTLs and the expression of GZMB in IgG4-RD patients. PBMCs from IgG4-RD patients (n = 19) were stimulated with 50 ng/mL IL-7 in the presence or absence of 150 nM tofacitinib for 4 days. **(A, D)** Representative dot plots of flow cytometry analysis. CD4+CD28-GZMB+ and CD8+CD28-GZMB+ CTL percentages in CD4 and CD8 T cells were shown. **(B, E)** The percentages of CD4+CD28-GZMB+ CTLs and CD8+CD28-GZMB+ CTLs in IgG4-RD patients were compared between IL-7-treated and untreated samples, and between IL-7-treated and IL-7 plus tofacitinib samples as well. The paired two-tailed Student’s t-test was used to compare two groups. ****P< 0.0001. **(C, F)** MFI of GZMB in CD4+CD28- CTLs and CD8+CD28- CTLs from IgG4-RD patients were compared between IL-7-treated and untreated samples, and between IL-7-treated and IL-7 plus tofacitinib samples as well. The paired two-tailed Student’s t-test was used to compare two groups. *P< 0.05; **P< 0.01; ***P< 0.001; ****P< 0.0001.

We analyzed the proportions of CD4+CD28-GZMB+ CTLs and CD8+CD28-GZMB+ CTLs and found that they were significantly increased after adding IL-7, but they recovered after adding JAK inhibitor tofacitinib (**Figures 6A, B, D, E**). We also analyzed GZMB+ cell percentages in CD4+CD28- CTLs and CD8+CD28- CTLs and found no significant changes after the addition of IL-7 (**Supplementary Figure 3**). Moreover, IL-7 increased the expression levels (MFI) of GZMB in CD4+CD28- CTLs and CD8+CD28- CTLs. And these effects also can be inhibited by tofacitinib (**Figures 6C, F**).

## Discussion

In this study, we systematically analyzed the changes of CD4+CD28- CTLs, CD8+CD28- CTLs, and their related subpopulations in peripheral blood of IgG4-RD patients, and then clarified the effects of IL-7 on the proliferation and subpopulation composition of CTLs and its mechanism. Our study demonstrated that IL-7 promotes the expansion and subpopulation composition of CD4+CD28- CTLs and CD8+CD28- CTLs through the JAK signaling pathway.

It is reported that CTLs play an important role in the pathogenesis of IgG4-RD (5, 16, 20–23). Our result revealed that the levels of CD28- CTLs were elevated in the circulation of IgG4-RD patients and IL-7 could significantly promote the expansion of CD28- CTLs. Our study has important implications for a more complete understanding of the role of CTLs in IgG4-RD. Especially, blockade of the IL-7 signaling pathway with a JAK inhibitor tofacitinib inhibits the expansion of CD28- CTLs, which indicates that tofacitinib may have potential therapeutic effects for IgG4-RD.

IgG4-RD is featured by often elevated levels of serum IgG4 and IgE (24–26). In line with previous studies, our findings also showed that the concentrations of serum IgG4 and IgE were significantly increased in IgG4-RD patients compared with HCs. The levels of these serum biomarkers are generally used as disease activity indicators in the therapy of this disease. Recently, researchers pay much more attention to circulating T cell subsets, which may be involved in the pathogenesis of IgG4-RD and can be used as biomarkers for monitoring the disease activity (27). Our previous study indicated that the levels of circulating Th1 and Tfh1 positively correlated with disease activity in IgG4-RD patients (28). CD4+ CTLs are mainly differentiated from Th1 cells (29). We further investigated the levels of CD4+CD28- CTLs and CD8+CD28- CTLs in IgG4-RD patients. According to our results, the levels of circulating CD4+CD28- CTLs and CD8+CD28- CTLs were increased significantly in IgG4-RD patients. Similarly, the levels of circulating CD4+CD28-CD127lo CTLs, CD8+CD28-CD127lo CTLs, CD4+CD28-GZMB+ CTLs, and CD8+CD28-GZMB+ CTLs were also significantly elevated in IgG4-RD patients. However, no significant correlations were found between CD28- CTL levels and disease activity or the concentrations of serum IgG4 and IgE. Our results indicated that circulating CD28- CTLs may be involved in the development of IgG4-RD and these CD28- CTL levels as a disease activity indicator should be evaluated with larger samples in future studies. A previous study showed that CD4+ CTLs and CD8+ CTLs play important roles in the pathogenesis of IgG4-RD by inducing apoptosis in disease lessions (5). Our study not only analyzed the changes of CTL subsets but also deeply analyzed the changes of related subsets characterized by CD127 and GZMB, which is helpful in accurately understanding the role of CD28- CTLs in IgG4-RD.

We focused our analysis on the causes of CTL elevation in IgG4-RD patients. IL-7 is involved in the pathogenesis of chronic inflammatory diseases and is targeted in patients with autoimmune diseases (30–32). Previously, several studies have stated that IL-7 was highly up-regulated and CD4+/CD8+ cytotoxic T cells were enriched in lesion tissue of patients with IgG4-RD (16, 17). According to our results, IL-7 drove the expansion of CD4+CD28- CTLs and CD8+CD28- CTLs in IgG4-RD patients. We also found that IL-7 promoted the expansion of CD28-CD127lo CTLs and CD28-GZMB+ CTLs in these patients. IL-7 should bind CD127 to act its effects. IL-7 induced CD28-CD127hi CTLs to down-regulate the expression of CD127 and meantime these CD28-CD127lo CTLs expanded. Previous studies have stated that IL-7 can reduce the expression of CD127 (15, 33). We used Ki67 to characterize the proliferation and found that IL-7 induced proliferation of CD4+CD28- CTLs and CD8+CD28- CTLs. We used MFI to characterize the expression levels GZMB and found that IL-7 promoted CD28- CTL cytotoxic function by up-regulating GZMB.

We dissected the mechanistic basis of IL-7 effects on CD4+CD28- CTLs and CD8+CD28- CTLs from IgG4-RD patients and demonstrated that tofacitinib, a selective JAK inhibitor blocking IL-7 signaling, significantly inhibited the expansion, proliferation, and function of these CTLs. Our results suggest that the IL-7/IL-7R/JAK pathway is an important molecular pathway for IL-7 to regulate CD28- CTLs in IgG4-RD, and our study proposes a molecular mechanism by which IL-7 regulates CTL levels.

Notably, our finding that IL-7 promoted CD28- CTL proliferation and function independently of antigen restimulation indicated that IL-7 may sustain the expansion and functions of these lymphocytes in the absence of T-cell receptor (TCR)-derived signals in IgG4-RD. A previous study stated that memory CD4+ T cells were promoted by either IL-7R or TCR signaling, whereas naive CD4 T cells require the cooperative activity of both of these receptor signaling pathways (34). So the expanded CD28- CTLs in our study should be memory T cells.

In particular, tofacitinib is a potent inhibitor of JAK1/JAK3 and inhibits JAK2 at high concentrations (35). Thus it can interfere with the activity of several cytokines in addition to IL-7. According to our results, we can only prove that tofacitinib can eliminate the changes in CTLs after adding IL-7, but it cannot be proved that tofacitinib only has an effect on the inhibition of IL-7. Objectively, tofacitinib may also affect CTLs in IgG4-RD patients by acting on cytokines other than IL-7, which needs to be studied in the future.

Our study still has many shortcomings, first of all, the sample size is not large enough, but it is enough to explain the effects of IL-7 upregulating JAK to promote the expansion of CD28- CTLs. We did not directly detect the JAK molecule, but only reflected its effect through the inhibitor because it was difficult for us to obtain enough CTLs for western blot detection through the limited blood routine remaining peripheral blood for cell sorting experiments. In addition, the current study did not address the clinical value of circulating CD28- CTL levels in the diagnosis of IgG4-RD patients, because it is difficult to collect a sufficient number of primary patients to elucidate its clinical value, which will be investigated in systematic research in the future.

In conclusion, in this study, we demonstrated the critical role of IL-7 in IgG4-RD, finding that administration of exogenous IL-7 could induce the expansion of CD4+CD28- CTLs and CD8+CD28- CTLs in IgG4-RD patients. The effects of IL-7 were inhibited by the administration of the JAK inhibitor tofacitinib. Therefore, IL-7 can affect the immune balance of IgG4-RD patients by promoting the expansion and function of CD4+CD28- CTLs and CD8+CD28- CTLs in IgG4-RD through the JAK pathway. Meanwhile, blocking the IL-7 signaling pathway has a potential therapeutic effect on IgG4-RD.

## Data Availability Statement

The raw data supporting the conclusions of this article will be made available by the authors, without undue reservation.

## Ethics Statement

The studies involving human participants were reviewed and approved by Ethics Committee of Peking University People’s Hospital. The patients/participants provided their written informed consent to participate in this study.

## Author Contributions

C-SX and CL designed the study; YL, AA, and XZ performed the experiments; YYL was responsible for subject recruitment; C-SX and CL wrote the paper. All the authors had approved the final draft submitted.

## Funding

This work was supported by grants from the National Natural Science Foundation of China (81871230), the Doctoral Fund of Ministry of Education of China (20120001120053), and Peking University People’s Hospital Scientific Research Development Funds (RDT 2020-01).

## Conflict of Interest

The authors declare that the research was conducted in the absence of any commercial or financial relationships that could be construed as a potential conflict of interest.

## Publisher’s Note

All claims expressed in this article are solely those of the authors and do not necessarily represent those of their affiliated organizations, or those of the publisher, the editors and the reviewers. Any product that may be evaluated in this article, or claim that may be made by its manufacturer, is not guaranteed or endorsed by the publisher.

## Supplementary Material

Supplementary Figure 1 The correlations of circulating CD4+CD28- CTL levels with IgG4-RD RI, serum IgG4 and IgE concentrations in IgG4-RD patients. Correlation analyses were conducted between the levels of CD4+CD28- CTLs and IgG4-RD RI **(A, D)** or the concentrations of serum IgG4 **(B, E)** and IgE **(C, F)** in IgG4-RD patients (n = 50). Each plot represented the data of one patient. The Spearman test’s correlation coefficient r and *P* values were listed, and the *P* values less than 0.05 were linearly regressed to show relevant trends.

Supplementary Figure 2 The correlations of circulating CD8+CD28- CTL levels with IgG4-RD RI, serum IgG4 and IgE concentrations in IgG4-RD patients. Correlation analyses were conducted between the levels of CD8+CD28- CTLs and IgG4-RD RI **(A, D)** or the concentrations of serum IgG4 **(B, E)** and IgE **(C, F)** in IgG4-RD patients (n = 50). Each plot represented the data of one patient. The Spearman test’s correlation coefficient r and *P* values were listed, and the *P* values less than 0.05 were linearly regressed to show relevant trends.

Supplementary Figure 3 The effects of IL-7 on the percentages of GZMB+ cells in CD28- CTLs from IgG4-RD patients. PBMCs from IgG4-RD patients (n = 19) were stimulated with 50 ng/mL IL-7 for 4 days. **(A, B)** The percentages of CD4+CD28-GZMB+ CTLs and CD8+CD28-GZMB+ CTLs in CD4+CD28- CTLs and CD8+CD28- CTLs from IgG4-RD patients were compared between IL-7-treated and untreated samples. The error bars represented the mean with SD. The paired two-tailed Student’s t-test was used to compare two groups. NS, not significant.

## Abbreviations

CTLs, cytotoxic T lymphocytes; IgG4-RD, IgG4-related disease; HCs, healthy controls; PBMCs, peripheral blood mononuclear cells; GZMB, granzyme B; IL-7, interleukin-7; JAK, Janus kinase; STAT, signal transducer and activator of transcription; MFI, mean fluorescence intensity.
