# Supplementary figures and images for: IL-7 Promotes the Expansion of Circulating CD28- Cytotoxic T Lymphocytes in Patients With IgG4-Related Disease via the JAK Signaling
Source: Front Immunol. 2022 Jul 7;13:922307. doi: 10.3389/fimmu.2022.922307 (PMC9301466; doi:10.3389/fimmu.2022.922307)

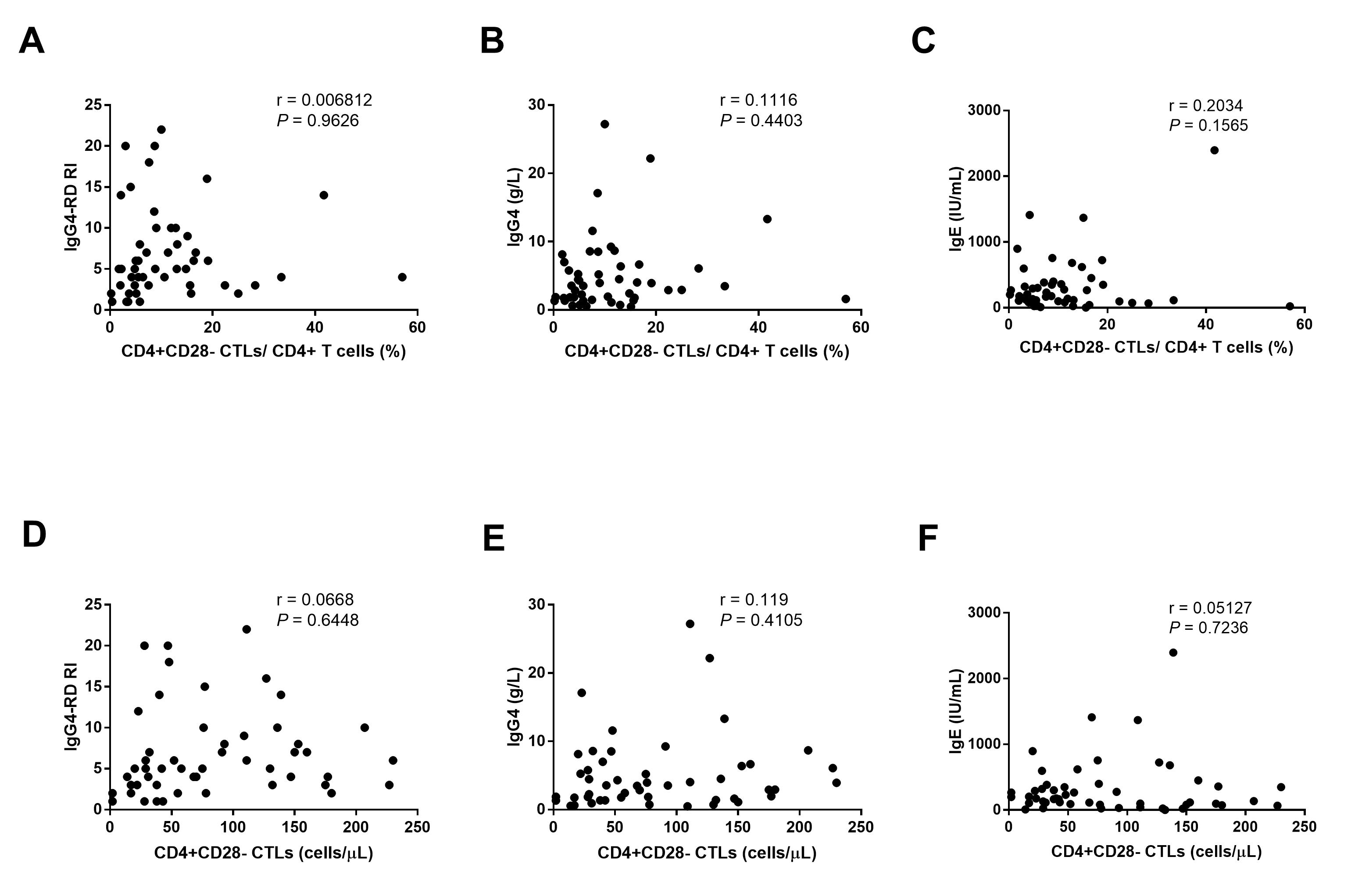

Supplement: Supplementary Figure 1 — The correlations of circulating CD4+CD28- CTL levels with IgG4-RD RI, serum IgG4 and IgE concentrations in IgG4-RD patients. Correlation analyses were conducted between the levels of CD4+CD28- CTLs and IgG4-RD RI (A, D) or the concentrations of serum IgG4 (B, E) and IgE (C, F) in IgG4-RD patients (n = 50). Each plot represented the data of one patient. The Spearman test’s correlation coefficient r and P values were listed, and the P values less than 0.05 were linearly regressed to show relevant trends. [file Image_1.jpeg]

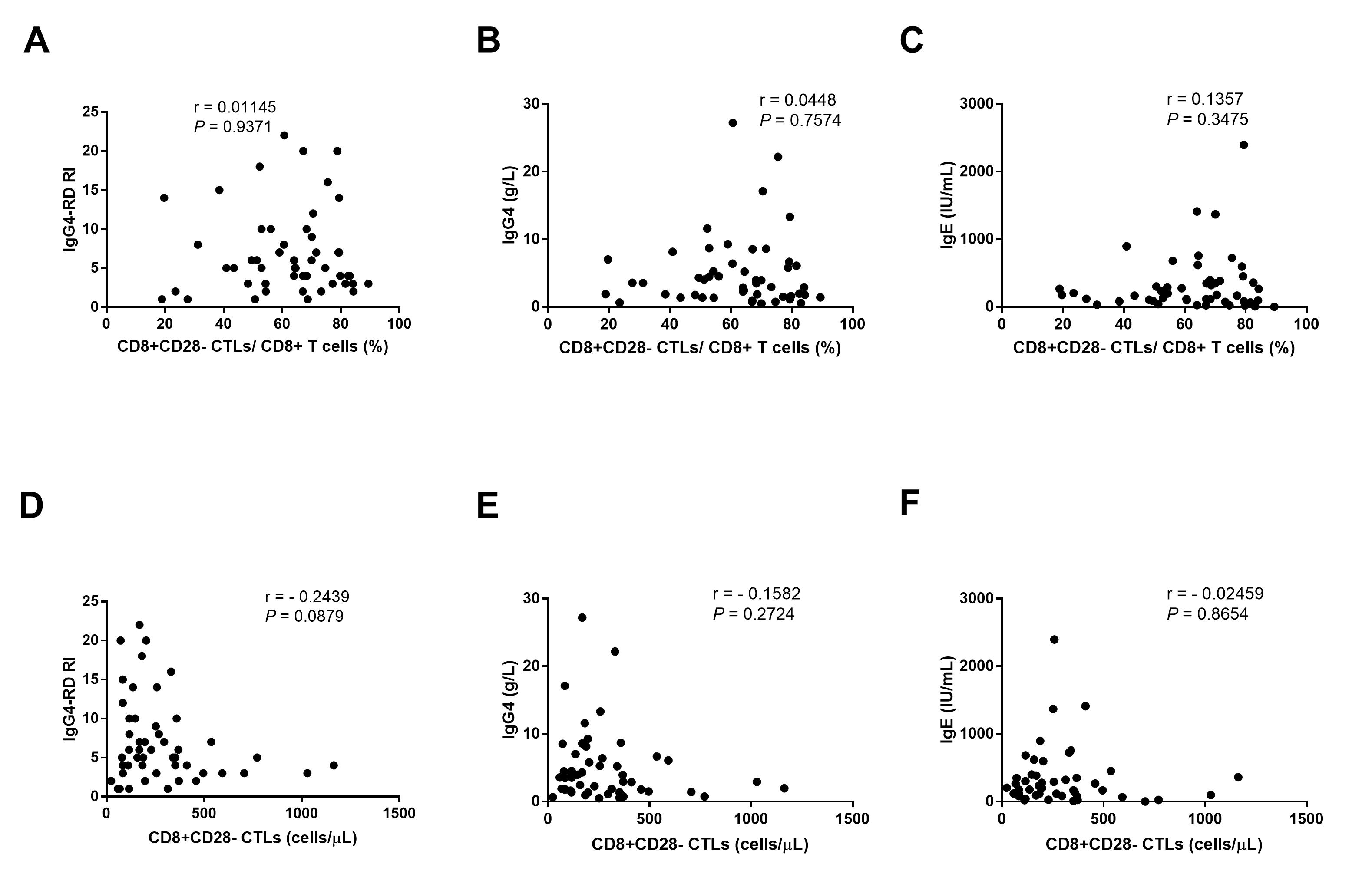

Supplement: Supplementary Figure 2 — The correlations of circulating CD8+CD28- CTL levels with IgG4-RD RI, serum IgG4 and IgE concentrations in IgG4-RD patients. Correlation analyses were conducted between the levels of CD8+CD28- CTLs and IgG4-RD RI (A, D) or the concentrations of serum IgG4 (B, E) and IgE (C, F) in IgG4-RD patients (n = 50). Each plot represented the data of one patient. The Spearman test’s correlation coefficient r and P values were listed, and the P values less than 0.05 were linearly regressed to show relevant trends. [file Image_2.jpeg]

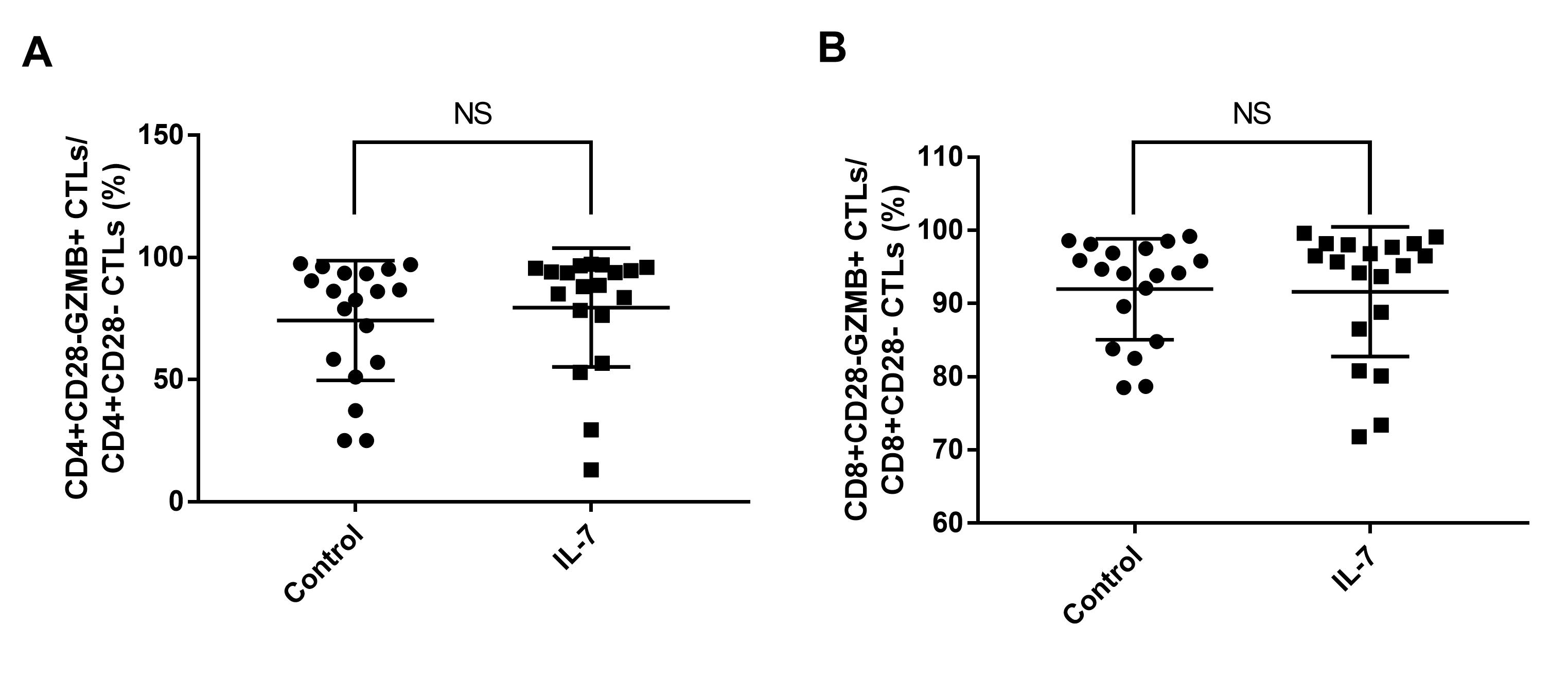

Supplement: Supplementary Figure 3 — The effects of IL-7 on the percentages of GZMB+ cells in CD28- CTLs from IgG4-RD patients. PBMCs from IgG4-RD patients (n = 19) were stimulated with 50 ng/mL IL-7 for 4 days. (A, B) The percentages of CD4+CD28-GZMB+ CTLs and CD8+CD28-GZMB+ CTLs in CD4+CD28- CTLs and CD8+CD28- CTLs from IgG4-RD patients were compared between IL-7-treated and untreated samples. The error bars represented the mean with SD. The paired two-tailed Student’s t-test was used to compare two groups. NS, not significant. [file Image_3.jpeg]
